# Simultaneous Protein Quantitation and Glycosylation Profiling of Antigen-Specific Immunoglobulin G1 in Large Clinical Studies

**DOI:** 10.1021/acs.jproteome.4c00538

**Published:** 2024-11-13

**Authors:** Steinar Gijze, Anna Wasynczuk, Leanne van Leeuwen, Marloes Grobben, Marit J. van Gils, Jan Nouta, Wenjun Wang, Virgil ASH Dalm, Hetty Jolink, Manfred Wuhrer, David Falck

**Affiliations:** † Center for Proteomics and Metabolomics, Leiden University Medical Center, 2300 RC Leiden, The Netherlands; ‡ Department of Viroscience, Erasmus University Medical Center, 3015GD Rotterdam, The Netherlands; § Department of Medical Microbiology and Infection Prevention, Amsterdam UMC Location AMC University of Amsterdam, 1105 AZ Amsterdam, The Netherlands; ∥ Department of Internal Medicine, Division of Allergy & Clinical Immunology; Department of Immunology, Erasmus University Medical Center, 3015GD Rotterdam, The Netherlands; ⊥ Department of Infectious Diseases, Leiden University Medical Center, 2300 RC Leiden, The Netherlands

**Keywords:** Glycoproteomics, antibody glycosylation, glycopeptides, liquid chromatography−mass spectrometry, LC-MS, stable isotope labeled protein standard, immunoglobulin G, antibody quantitation, antigen-specific antibody responses

## Abstract

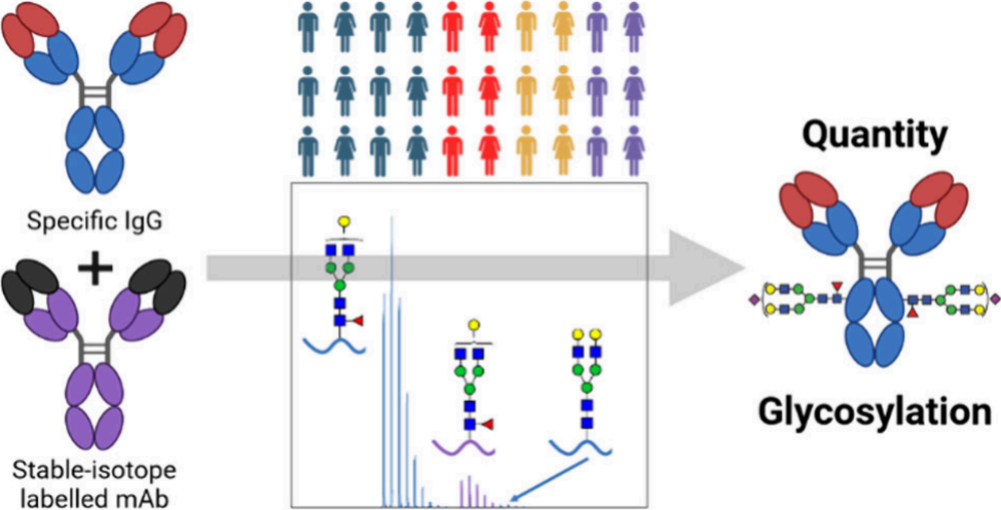

Antibodies have a key role in the immune system, making their characterization essential to biomedical, biopharmaceutical, and clinical research questions. Antibody effector functions are mainly controlled by quantity, subclass, and Fc glycosylation. We describe an integrated method to measure these three critical dimensions simultaneously. The subclass-specific immunoglobulin G (IgG) Fc glycosylation analysis combines immunosorbance with glycopeptide-centered LC-MS detection. For integrated IgG1-specific quantitation, a commercial, stable isotope labeled IgG1 protein standard was spiked into the immunosorbent eluates. Robust quantitation was achieved, relying on a combination of a proteotypic peptide and the most abundant glycopeptides, generated through proteolytic cleavage from a mixture of natural IgG1 and the recombinant IgG1 standard. Method performance was demonstrated in a large coronavirus vaccination cohort at a throughput of 100 samples/day. LC-MS-derived, anti-SARS-CoV-2 spike protein IgG1 concentrations ranged from 100 to 10000 ng/mL and correlated well with a clinically relevant immunoassay. Technical variation was 200 times lower than biological variation; intermediate precision was 44%. In conclusion, we present a method capable of robustly and simultaneously assessing quantity, subclass, and Fc glycosylation of antigen-specific IgG in large clinical studies. This method will facilitate a broader understanding of immune responses, especially the important interplay among the three dimensions.

## Introduction

Antibodies are key immune molecules bridging adaptive and innate immunity through the translation of antigen recognition into Fc receptor-mediated immune cell activation.^1^ The activation of effector functions by IgG is controlled through a complex dependency on concentration, subclass use, and glycosylation.^2,3^ Therefore, a method that can assess all three aspects and their interdependencies simultaneously is of great value for immunological and clinical research. In recent years, an increasing number of studies has demonstrated the highly desirable integration of these data, but the individual aspects have been assessed by different techniques.^4−6^ First, this hinders studies by requiring a range of different expertise, often from different laboratories. Second, some interdependencies, such as subclass-specific glycosylation or relative subclass abundances, might be lost this way.

IgG glycosylation analysis is often performed with instrumental techniques, most commonly combining a separation technique with either fluorescence or mass spectrometry detection.^7^ In contrast, IgG quantities are mostly derived from biochemical assays, prominently enzyme-linked immunosorbent assays (ELISA), UV-adsorption measurements, or colorimetric assays. We have previously established GlYcoLISA, a protocol for analysis of antigen-specific antibody glycosylation using a combination of immunosorbent purification and liquid chromatography–mass spectrometry (LC-MS) detection.^8^ GlYcoLISA covers a wide variety of glycosylation traits, including effector-function relevant fucosylation, bisection, sialylation, and galactosylation, but also individual glycoforms, since the readout is MS-based. As it is a technological hybrid between techniques commonly used for the assessment of glycosylation and quantities of IgG, it is a uniquely suited starting point to integrate their assessment into a single assay. We achieved this through the implementation of a stable isotope labeled (SIL) protein standard into our glycopeptide-centric workflow.

Absolute quantitation of proteins by LC-MS is typically achieved using stable isotope labeling.^9,10^ When analyzing whole proteomes of an organism or tissue, chemical or metabolic labeling techniques, such as TMT-labeling or SILAC, are often applied.^10,11^ However, for simpler tasks like the herein attempted quantitation of a single protein or protein family, the combination of label-free quantitation and SIL standards offers a more efficient alternative.^9^ SIL peptide standards, so-called AQUA peptides,^12^ are readily available through peptide-synthesis facilities, but SIL glycopeptide standards are largely lacking.^7^ Alternatively, SIL glycoprotein standards may be used, but they are equally scarce. While increasingly applied in structural characterization by nuclear magnetic resonance, examples of the use of SIL glycoprotein standards in MS-based quantitation are still rare.^13,14^ Fortunately, the large commercial interest in IgG as a biologic in the form of therapeutic monoclonal antibodies has led to the commercial availability of SIL IgG standards. SIL protein standards are added during sample preparation.^14^ Thus, they experience the same sample preparation steps as the natural proteins, importantly purification and proteolytic cleavage, and can thus be used to correct for systematic and random errors in these steps. The natural and SIL (glyco)peptides obtained through standard bottom-up workflows are then simultaneously analyzed. They have different masses but very similar physicochemical properties, which further allows for correction of errors, importantly those caused by variations in ionization efficiency.

IgG responses, including their glycosylation, have recently commanded much attention in the study of the COVID-19 pandemic and in its management through vaccination campaigns.^5,15^ A number of COVID-19 vaccines are available, including the mRNA (mRNA) vaccine developed by Moderna.^16^ The mRNA encodes for the spike protein of severe acute respiratory syndrome coronavirus 2 (SARS-CoV-2) that is translated mainly by hepatocytes of the vaccinee thus raising an antispike (anti-S) immune response.^17^

We introduce here a method to simultaneously obtain subclass-specific IgG glycosylation profiles as well as IgG abundances, which is based on combining the GlYcoLISA protocol with a SIL IgG standard. Precision and accuracy were assessed by repeated measurements of a pooled sample and comparison to an established method which has been validated for large clinical studies.^18^ We chose to demonstrate the performance of our method in a large clinical study, namely, the VACOPID (Vaccination Against COvid in Primary Immune Diseases) study encompassing a total of about 700 healthy volunteers and patients with inborn errors of immunity (IEI), in order to demonstrate not only its general ability to measure antibody quantities but also its robust applicability to such a challenging scenario.

## Experimental Section

### Samples and Materials

Plasma samples from the VACOPID cohort study were obtained for 516 out of approximately 700 participants from Erasmus Medical Center (EMC), Leiden University Medical Center (LUMC), Maastricht University Medical Center (MUMC), University Medical Center Groningen (UMCG), and University Medical Center Utrecht (UMCU).^19^ The VACOPID study is a prospective, controlled, multicenter study performed among patients with IEI from 7 academic hospitals in The Netherlands. The study adhered to the principles of the Declaration of Helsinki and was approved by the Dutch Central Committee on Research Involving Human Subjects (CCMO, NL7647.078.21, EudraCT number 2021-000515-24), the Medical Research Ethics Committee from Erasmus University Medical Center (MEC-2021-0050), and the local review boards of all other participating centers. All participants provided written informed consent before enrollment. The participants in this cohort were vaccinated against COVID-19, using the Moderna mRNA (mRNA) vaccine. Blood was drawn twice, 28 days after the first and second vaccination. Of these, 933 samples were analyzed for 516 participants. Twenty-eight plasma samples from patients with X-linked agammaglobulinemia (XLA) were used as negative control sample. Aliquots of 20 to 30 different samples (per hospital, depending on required volume) were pooled to generate samples for replicate measurements.

### Assessment of anti-S IgG Levels by Luminex

A custom Luminex assay was used to measure IgG antibody levels to the prefusion-stabilized trimeric SARS-CoV-2 spike (S) protein, as described previously.^18^ The prefusion 2P S ectodomain (residues 1–1138, Wuhan-Hu-1, GenBank MN908947.3) protein of SARS-CoV-2 was produced in HEK293F cells (ThermoFisher) and purified by affinity purification using Ni-NTA agarose beads.^20^ The protein was covalently coupled to Luminex Magplex beads using a two-step carbodiimide reaction as previously described.^18^

To measure the binding of IgG to the S protein, 100.000-fold serum dilutions were mixed with the protein coupled beads and incubated overnight on a rotator at 4 °C. The next day, plates were washed, incubated with Goat-antihuman IgG-PE (Southern Biotech) for 2 h and read-out was performed on a Magpix (Luminex).^18^ The WHO International Standard for anti-SARS-CoV-2 immunoglobulin (NIBSC 20/136) was used to convert the median fluorescence intensity (MFI) output into binding antibody units per ml (BAU/ml).

### Purification of anti-Spike Antibodies and LC-MS (Glyco-)peptide Measurements

Anti-S IgG was captured from 20 μL of the plasma samples using affinity purification as described in our GlYcoLISA protocol.^8^ Spike protein (antigen) was immobilized on a 96-well plate, incubated with plasma, and washed. Anti-Spike IgG was eluted with 100 μL of 100 mM formic acid spiked with the SILuMAB IgG1 protein standard (0.02 ng/μL). Each sample eluate thus contained 2 ng of SILuMAB, equivalent to 100 ng/mL of IgG1 in plasma. Tryptic cleavage of anti-S IgG and LC-MS (glyco-)peptide measurements were also carried out following the GlYcoLISA protocol.^8^ In brief, (glyco-)peptides were separated on a NanoEase M/Z peptide BEH C18, 1.7 μm particles 130 Å pores, 75 μm × 100 mm (Waters) at a flow rate of 600 nL/min using a binary gradient of 0.02% aqueous TFA and 95% acetonitrile. The nanoLC-ESI-MS setup combined an Ultimate 3000 LC (Thermo Fisher Scientific) with an Impact time-of-flight MS (Bruker Daltonics) via a CaptiveSpray source using acetonitrile-enriched nitrogen as drying gas. Fragmentation was performed by targeting specific precursor ions and using stepping-energy collision-induced dissociation at 45, 50, and 70 eV.

### Data Processing and Curation

MS^1^ signals of (glyco-)peptides were quantified by integration of the MS peak area after generation of sum spectra of the retention times of interest.^8,21^ Raw LC-MS data files were converted to mzXML files using ProteoWizard MSConvert (version 3.0.8708). The mzXML files were processed in LaCyTools version 2.0.1 (build 20201216), largely following the GlYcoLISA protocol, but with some deviations.^8,21^ In addition to the natural IgG1 glycopeptides mentioned in the GlYcoLISA protocol, the three glycopeptides resulting from tryptic cleavage of SILuMAB (IgG1-G0, -G0F and G1F, considering [M+2H]^2+^ and [M+3H]^3+^) were used as potential calibrants. A signal-to-noise (S/N) cutoff of 9 was used for the calibrants. Integration was performed by using a mass window of 0.04 Th around each isotopologue peak. The minimum fraction of the integrated isotopologue pattern was set to 0.95. Nonglycosylated peptides were quantified as [M+2H]^2+^ without calibration, using an integration mass window of 0.065 Th and minimum isotopologue fraction of 0.8.

We performed a spectral curation based on the negative controls. For all samples and controls, the ratio between the total spectrum intensities of natural anti-S IgG1 and SILuMAB was calculated. The 95th percentile of these ratios in the negative controls was then taken as a cutoff, below which samples were excluded from further analysis. Of the 933 samples, 844 were selected for the method comparisons as they could be quantified by both Luminex and LC-MS.

An analyte consensus list was created for the natural anti-S IgG1 glycopeptides based on the four largest biological groups in the LUMC samples, using a cutoff of 80% as described in our GlYcoLISA protocol.

### Quantitation of anti-Spike IgG1

Abundances of each glycopeptide or peptide were calculated by summing all charge states and isotopes and correcting for the isotopologue coverage. Per sample, abundances of all anti-Spike IgG1 glycopeptides and separately of all SILuMAB glycopeptides were summed. Anti-S IgG1 concentrations in plasma were then calculated based on their ratios as follows:
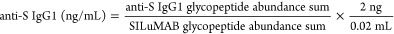
1The second term relates the calculated concentrations to 20 μL (0.02 mL) of plasma, from which anti-S IgG1 was captured, and 2 ng of SILuMAB was added.

Anti-S IgG1 quantitation, based on the proteotypic peptide GPSVFPLAPSSK (GPS), was performed using the ratio between the corrected intensities of the anti-Spike IgG1 and SILuMAB peptide:
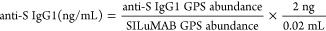
2For each sample, the anti-S IgG1 concentration was calculated as the median of the concentrations derived from the glycopeptides and the GPS peptide.

### Statistics

All correlation plots were assessed with Spearman rank correlation, providing correlation values (*r*) and a significance (*p*) value. Otherwise, only descriptive statistics were reported.

## Results and Discussion

**Figure 1:**
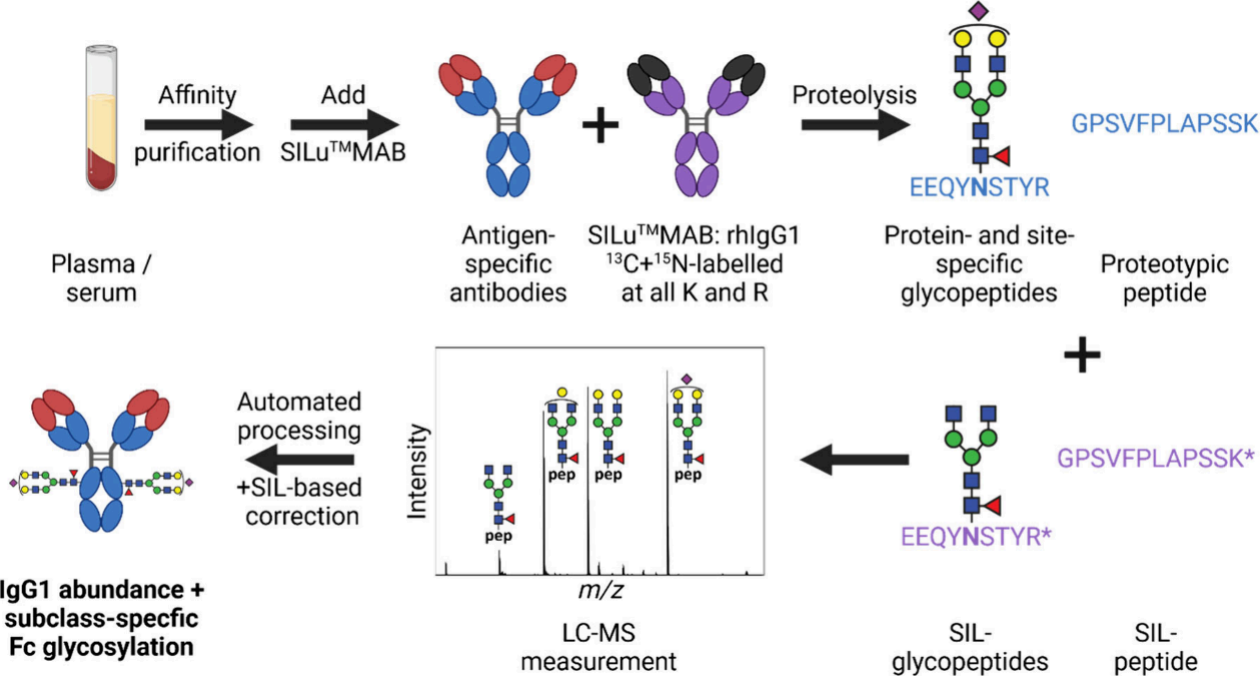
Workflow for simultaneous quantitation and Fc glycosylation analysis of antigen-specific IgG. For correction of measurement variability, a stable-isotope labeled (SIL) human recombinant IgG1 (rhIgG1), commercially available as SILuMAB, was used.

**Figure 2:**
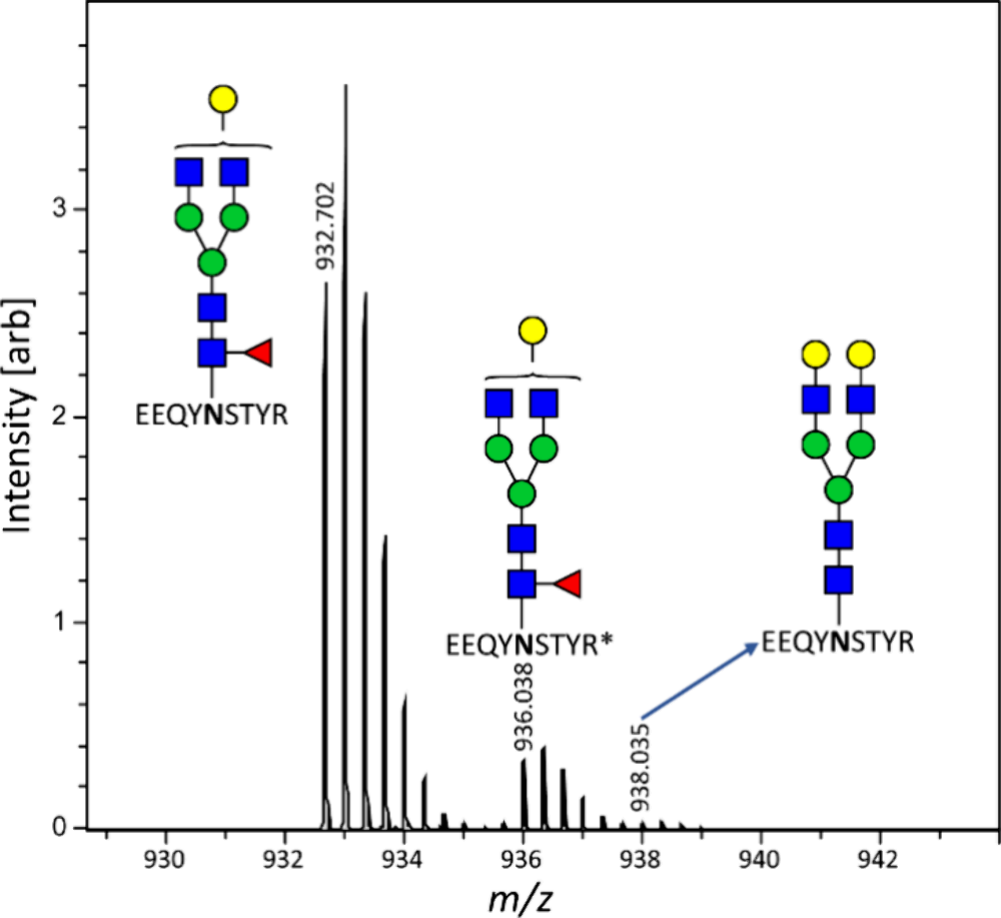
Zoomed-in view of a sum spectrum of the IgG1 elution range (48 to 62 s). The isotopologue pattern of the heavy labeled IgG1-G1F glycopeptide of SILuMAB is well-separated from that of the natural glycopeptide. Importantly, it also does not interfere with the far-less abundant IgG1-G2 glycopeptide. R* = heavy labeled arginine.

Based on our subclass-specific IgG glycosylation analysis protocol, GlYcoLISA,^8^ we explored the possibility to integrate absolute IgG1 quantitation using a stable isotope labeled monoclonal antibody (SILuMAB; Figure 1). SILuMAB is labeled with ^13^C and ^15^N at all lysines and arginines, resulting in isotopologues of tryptic (glyco)peptides with 8 or 10 additional neutrons compared to the naturally occurring monoisotopic molecules. As shown in Figure 2, the resulting 10 Da mass difference puts the isotopologue pattern of the heavy labeled fucosylated glycopeptides perfectly between the patterns of the fucosylated and afucosylated forms of the natural glycopeptides. Thus, quantitation of afucosylation is maintained in our approach, which is an important feature, as afucosylation is one of the most functionally impactful glycosylation traits.^5^

Adding a generic mAb, such as SILuMAB, after capturing makes the protocol broadly applicable to investigations of any antigen specificity as opposed to adding an mAb of the specificity under investigation to plasma directly. However, capturing efficiency is not taken into account, resulting in an underestimation compared to the true plasma concentration. Nonetheless, since the capturing is part of an established ELISA assay,^22^ variability in capturing efficiency will be largely biological (varying affinities of the polyclonal response), not technical.

**Figure 3:**
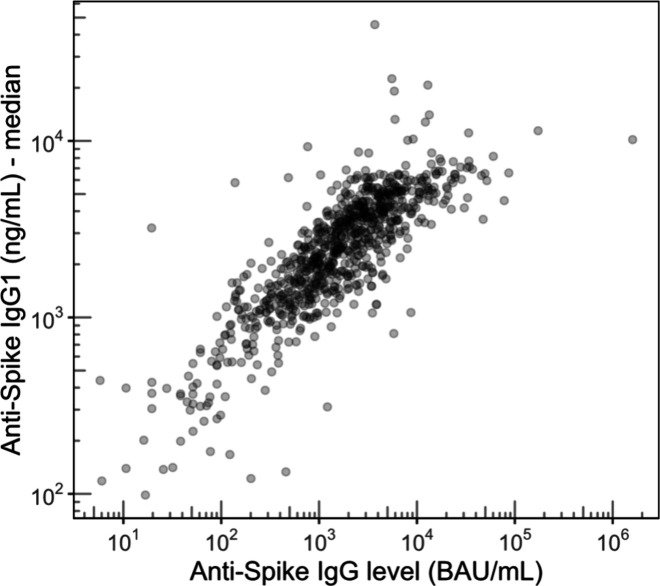
GlYcoLISA anti-S IgG1 concentrations showed a high correlation with anti-S IgG Luminex levels. Only samples yielding a value in both methods were included (Figure S1). Spearman rank correlation *r* = 0.83 (*p* < 0.001). *n* = 844. BAU = Binding Antibody Units.

As the final value for anti-S IgG1 concentrations determined by our approach, we used the median of the concentration derived from two tryptic peptides (glycopeptides and GPS peptide; see the explanation below). It is important to realize that the absolute values of the anti-S IgG1 quantitation are based on gravimetric methods and the fundamental chemical equality of isotopologues and, as such, do not require validation. Due to the inability to correct for capturing efficiency, accuracy should be interpreted relatively for this assay and has a lower relevance than precision. In order to estimate the precision of our quantitation, we compared our values to levels obtained with an established method, namely, an anti-S IgG Luminex assay, which has been extensively validated and successfully applied to large clinical cohorts including VACOPID (Figures 3 and S1).^18^ XLA patients cannot produce antibodies and therefore were also negative for anti-S IgG in the Luminex assay. Consequently, we used these patients’ samples to determine the unspecific background signal of our LC-MS assay and exclude measurements in which an equal or lower concentration was calculated. Comparison between our quantitation method and the Luminex assay has to be interpreted with care due to fundamental assay differences resulting in different, noninterconvertible units. Nonetheless, the results are highly consistent with the established method (*r* = 0.83) validating that our method is capable of reliably quantifying anti-S IgG1. The lower and upper limits of quantitation were 100 and 10000 ng/mL, respectively (Figure 3).

GlYcoLISA offers reliable relative quantitation of IgG1 glycopeptides. Therefore, we first assessed absolute IgG1 quantitation based on the glycopeptides. SILuMAB, being a CHO cell produced IgG1 mAb, showed a typically narrow distribution of glycoforms (Figure S2) that differed strongly from the glycoform distribution of the natural IgG.^7^ We restricted coverage to the three most abundant glycoforms of SILuMAB, G0, G0F, and G1F, which gave well-quantifiable signals at 2 ng of SILuMAB, thus minimizing consumption of the expensive protein standard. These were observed at the following *m*/*z* values (exact mass of first isotopologue for [M+2H]^2+^ and [M+3H]^3+^) and retention times: GO *m*/*z* 833.337, *m*/*z* 1249.502 and 88 s; G0F *m*/*z* 882.023, *m*/*z* 1322.531 and 85 s; G1F *m*/*z* 936.041, *m*/*z* 1403.557 and 83 s. We estimate that these amount to ca. 84% of the total glycoprofile. Their sum abundance was consequently used to represent SILuMAB in the calculations based on the glycopeptides (eq 1). For the natural IgG1, the 20 glycopeptides listed in Table S1 were quantified and their sum used to calculate absolute IgG1 concentrations (glycoprofile coverage >95%). The glycoform differences between the natural IgG1 and SILuMAB were a potential concern with respect to correcting variability, for example in ionization efficiency.

To achieve a more robust quantitation, we aimed to include additional independent analytes as a basis for the quantitation. Using multiple analytes for quantitation allows monitoring of interferences, which will be specific to one *m*/*z* and retention time combination, and reduces their impact on quantitation outcomes.^23,24^ This is already partially achieved by summing the three glycopeptides of SILuMAB. We found that we could additionally quantify two tryptic peptides without a glycosylation site. These were identified as the proteotypic GPSVFPLAPSSK (GPS) and TTPPVLDSDGSFFLYSK (TTP), a peptide that is unique for IgG1 only within the immunoglobulin family, as shown in Figure S3. Though it was possible to use TTP for IgG1 quantitation, results did not correlate as well with the levels (*r* = 0.69; Figure S4) as for the quantitation based on the glycopeptides (or on GPS), likely due to significant interfering signals. The better performance of GPS is in line with its greater specificity within the human proteome and the limits of purity that can be expected from the affinity purification. Thus, TTP quantitation results were not used in the final composite concentration. In contrast, the proteotypic GPS peptide performed well enough in the quantitation, showing a correlation score closer to that of the glycopeptide-based quantitation (*r* = 0.76 versus *r* = 0.84; Figure S5). GPS SILuMAB signals were also remarkably stable over time (RSD = 38%; Figure S1B). Importantly, results from glycopeptide- and GPS-based quantitation correlated very well with each other (*r* = 0.88) as shown in Figure S6. TTP-based quantitation results did not correlate as well with those based on the other analytes (*r* = 0.81 and *r* = 0.69 for TTP versus glycopeptides and GPS, respectively). The glycopeptide-based quantitation seems to have a smaller pseudolinear range than the GPS-based one (Figure S6). Within the linear range (up to ∼10^4^ ng/mL), the quantitation based on the glycopeptides gives higher concentrations compared to the quantitation based on the GPS peptide.

The independently good correlation of the glycopeptide- and GPS-based anti-S IgG1 concentrations with the anti-S IgG Luminex levels as well as the high correlation between the concentrations further supports a high accuracy of our quantitation method. The use of multiple analytes for quantifying anti-S IgG1 makes our method more robust against outliers.

Technical variation in observed quantities of IgG1 is about a factor of 4, which includes batch effects of capturing efficiency (Mean ± SD 5.4 ± 2.4 μg/mL, coefficient of variation 44%, 19 repeated measurements; Figure S7). In contrast, the IgG1 concentrations measured in the VAOCPID cohort span a factor of about 100. This allows a reliable assessment of biological effects, as these are largely the cause of the measured variation in the anti-S IgG1 concentrations.

**Figure 4:**
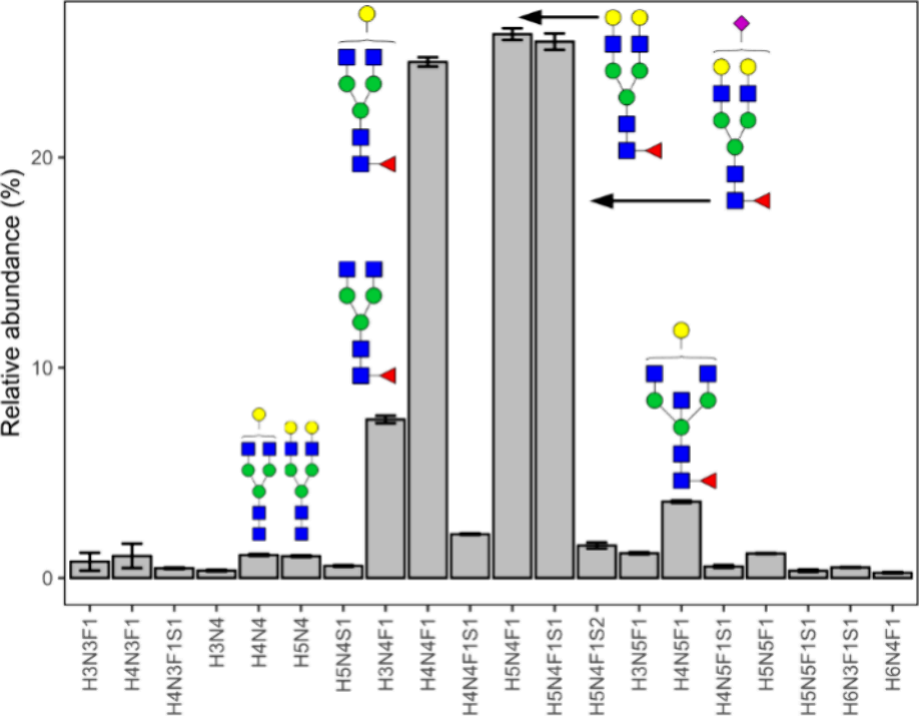
Glycosylation profiles of anti-S IgG1 from replicate measurements of the pooled sample in several batches of the EMC hospital samples (*N* = 19). The low variability and the good fit with expectation values underline that SILuMAB addition does not disturb glycosylation measurements. Shown are mean and standard deviation. Cartoons show tentative glycan structures of selected, abundant glycoform.

Importantly, the addition of SILuMAB did not disturb the assessment of the glycosylation of the natural IgG. Anti-S IgG1 and IgG3 profiles were highly precise, as illustrated by the replicate measurements of a sample pool over several batches shown in Figure 4. Furthermore, the obtained glycosylation profiles were in line with observations in previous studies, showing the same major glycoforms and comparable amounts of galactosylation, sialylation, bisection and fucosylation.^25^

Next to IgG1, anti-S IgG3 responses were observed in the subjects of the VACOPID cohort. Since GlYcoLISA is able to assess IgG1 and IgG3 separately, we investigated whether the IgG1 SILuMAB was suitable to serve as a standard for IgG3 quantitation as well (Figure S8). Although some sources of variability, such as ionization suppression at the specific retention time, cannot be corrected for, others, such as proteolytic cleavage efficiency and general instrument performance, would be expected to impact IgG1 and IgG3 in a similar manner. We quantified anti-S IgG3 based on the glycopeptide ratios, using G0F, G1F, G1FS, G1FN, G2F, and G2FS for IgG3 (Figure S8A). In contrast to anti-S IgG1, we cannot assess whether the quantitation of anti-S IgG3 is successful because we have no specific reference values for IgG3 concentrations. Since the total anti-S IgG responses, and thus the Luminex values, are dominated by IgG1 in almost all samples, the poor correlation between the MS-determined IgG3 concentrations and the Luminex levels (Figure S8A; *r* = 0.49) is consistent with a similar correlation between our IgG1 and IgG3 concentrations (Figure S8B; *r* = 0.54). This poor correlation may have both technical and biological origins. Comparing the sum of IgG1 and IgG3 concentrations to the Luminex levels (Figure S8C), neither improves nor worsens the correlation, thus not providing any further insights into the matter.

## Conclusions

We present a method that allows for the simultaneous glycoprofiling and quantitation of IgG1. Having a method that can simultaneously assay the three key parameters (quantity, subclass, and glycosylation) will greatly streamline research into antigen-specific antibody responses in infection, immunity, and therapeutic interventions. Based on the SIL protein standard SILuMAB, our quantitation method is reasonably robust and precise, with results correlating well with an established quantitation technique. Robustness is strengthened by the combined use of glycopeptides and a proteotypic peptide for quantitation. Importantly, the precision and accuracy of the glycoprofiling remained as good as those of the original GlYcoLISA protocol. We further demonstrated that the performance of the presented approach is sufficient to be applied to the analysis of large clinical cohorts.

The important clinical results of our investigation were out of the scope of this article but will be described elsewhere in the future. Further research is needed as to whether the other IgG subclasses can be quantified based on the IgG1 SIL standard or if they need either additional SIL peptides or individual SIL protein standards. Recently, we have integrated the SIL-based quantitation into our data curation application GlycoDash (https://github.com/Center-for-Proteomics-and-Metabolomics/glycodash) which allows performing the presented calculations automatically.

## Supporting Information Available

Supporting figures and tables containing structural assignments, variability data, and additional correlation plots (PDF)
