# Supplementary material for: Simultaneous Protein Quantitation and Glycosylation Profiling of Antigen-Specific Immunoglobulin G1 in Large Clinical Studies
Source: J Proteome Res. 2024 Nov 13;23(12):5600–5. doi: 10.1021/acs.jproteome.4c00538 (PMC11629375; doi:10.1021/acs.jproteome.4c00538)
Supplement: Supplementary file 1 — pr4c00538_si_001.pdf [file pr4c00538_si_001.pdf]

Simultaneous protein quantitation and glycosylation profiling of antigen-specific immunoglobulin G1 in large clinical studies

Steinar Gijze[1], Anna Wasynczuk[1], Leanne van Leeuwen[2], Marloes Grobben[3], Marit J. van Gils[3], Jan Nouta[1], Wenjun Wang[1], Virgil ASH Dalm[4], Hetty Jolink[5], Manfred Wuhrer[1], David Falck[1,*]

[1] Center for Proteomics and Metabolomics, Leiden University Medical Center, Leiden, The Netherlands
[2] Department of Viroscience, Erasmus University Medical Center, Rotterdam, The Netherlands
[3] Department of Medical Microbiology and Infection Prevention, Amsterdam UMC location AMC University of Amsterdam, Amsterdam, The Netherlands
[4] Department of Internal Medicine, Division of Allergy & Clinical Immunology; Department of Immunology, Erasmus University Medical Center, Rotterdam, The Netherlands
[5] Department of Infectious Diseases, Leiden University Medical Center, Leiden, The Netherlands

*d.falck@lumc.nl

Table of contents:

Table S1: compositions, names and structures of IgG1 glycans S-2
Figure S1: Correlation of anti-Spike IgG1 concentrations with anti-Spike IgG Luminex® levels S-3
Figure S2: Variability in the signal of the SIL (glyco-)peptides S-4
Figure S3: Identification of IgG1 proteotypic peptide S-5
Figure S4: Anti-S IgG1 concentrations based on TTP S-6
Figure S5: Anti-Spike IgG1 concentrations (Glycopeptides and GPS separately) S-6
Figure S6: Correlation of utilized (glyco-)peptides for anti-Spike IgG1 concentrations S-7
Figure S7: Anti-Spike IgG1 concentrations (ng/mL) in the EMC pool samples S-8
Figure S8: IgG3 quantitation S-9

Table S1: Compositions, names and structures of IgG1 glycans.

Glycan composition	Name	Structure*	GlyTouCan structure ID*
H3N3F1	G0F-N	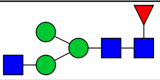	G07483YN
H4N3F1	G1F-N	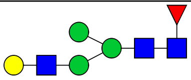	G12398HZ or
H4N3F1S1	G1FS-N	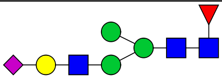	G30109UP
H3N4	G0	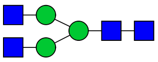	G39213VZ
H4N4	G1	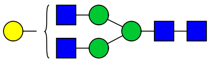	G66937TJ and G89993FE
H5N4	G2	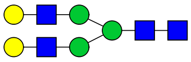	G36191CD
H5N4S1	G2S	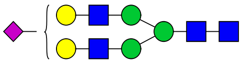	G94239KE (sialic acid is α2-
H3N4F1	G0F	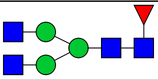	G80858MF
H4N4F1	G1F	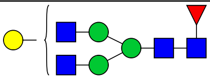	G58667NI and G27919IH
H4N4F1S1	G1FS	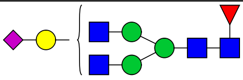	G43694RQ or G23295TF
H5N4F1	G2F	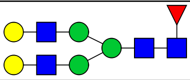	G78059CC
H5N4F1S1	G2FS	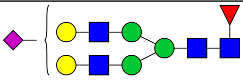	G81413UE
H5N4F1S2	G2FS2	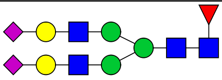	G56749GV
H3N5F1	G0FN	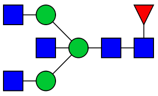	G30159WR
H4N5F1	G1FN	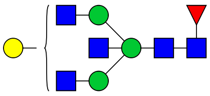	G85767HW or G71013KY
H4N5F1S1	G1FNS	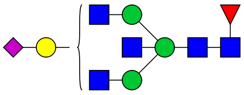	G39472EX
H5N5F1	G2FN	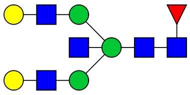	G25520XG
H5N5F1S1	G2FNS	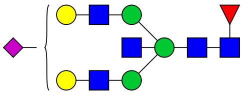	G18422YU (sialic acid is α2-6)
H6N3F1S1	Man5G1FS hybrid	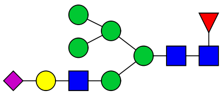	G93107BF
H6N4F1	Man5G1FN hybrid	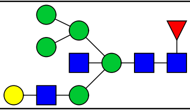	

*We assumed that 1) all structures have a normal N-glycan core, 2) the first GlcNAc modifies the α1-3 arm, 3) linkages are β2, β4 and α6 for GlcNAc, Gal and Sia, respectively, and 4) the third GlcNAc is a bisecting GlcNAc.

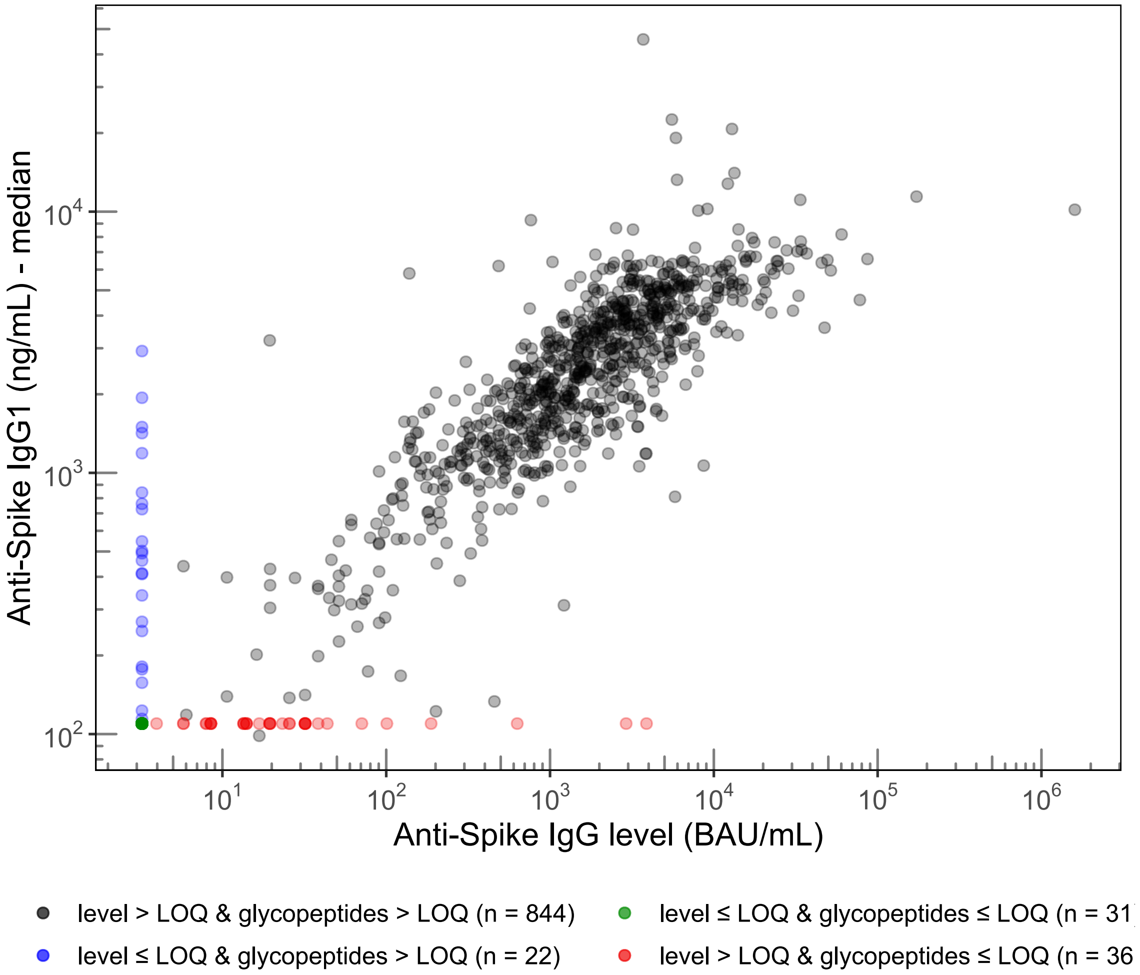

Figure S1: Correlation of anti-Spike IgG1 concentrations with anti-Spike IgG Luminex® levels. This figure includes samples for which the level was at the limit of quantitation (LOQ = 3.23 BAU/mL), and/or for which the sum spectra failed curation. Samples whose sum spectra failed curation are assigned a concentration of 109.7 ng/mL, which is equal to the LOQ of our method. r = 0.86 (p < 0.001). n = 933.

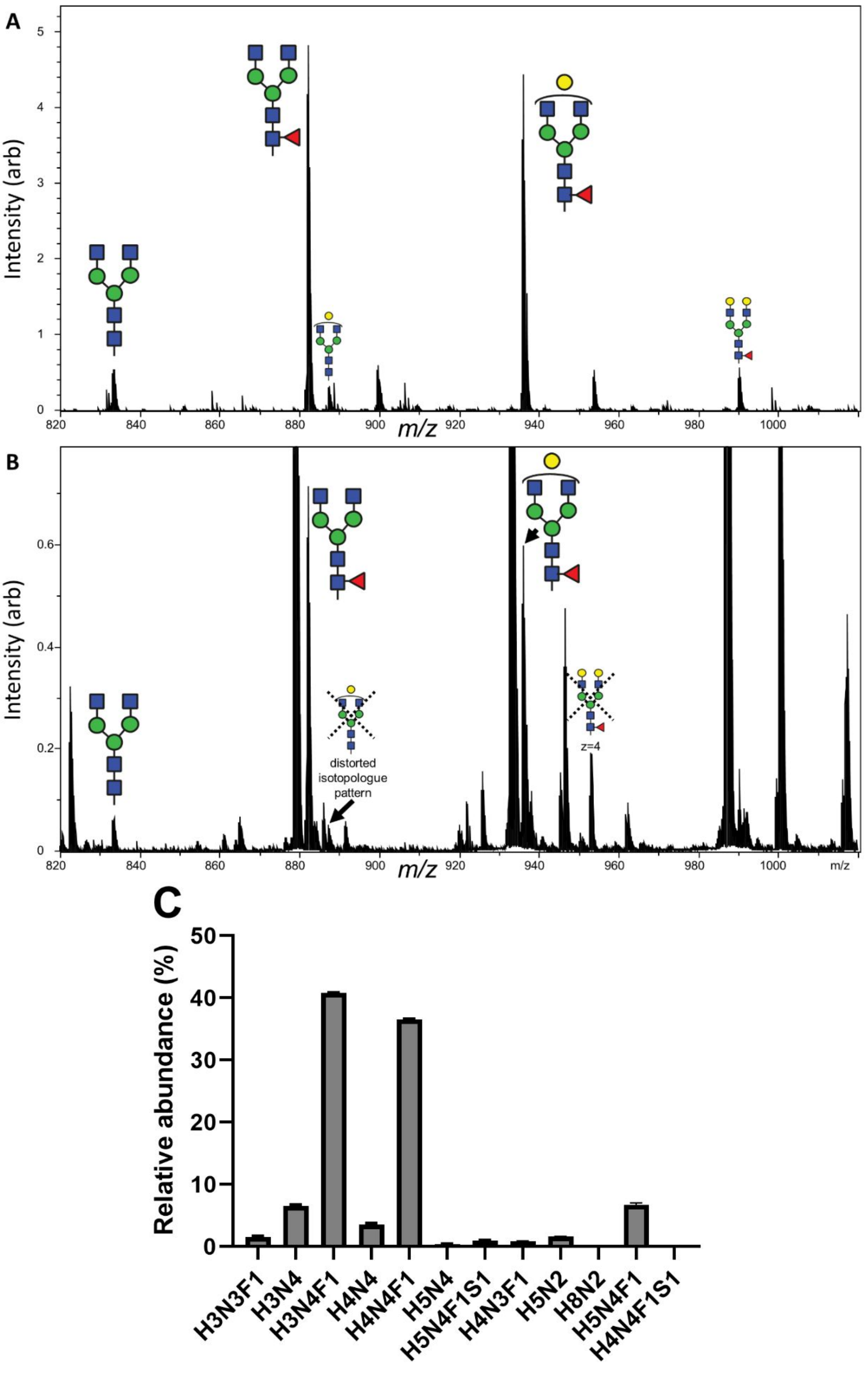

Figure S2: Characterization of SILu™MAB glycosylation. A) Annotated sum spectrum of direct measurement (mass range of $[M+3H]^{3+}$-ions). B) Annotated sum spectrum of sample with spiked SILu™MAB (mass range of $[M+3H]^{3+}$-ions; Zoom to intensity range of SILuMAB). Crossed out structures of G1 and G2F indicate that these SILuMAB glycopetides could not be quantified in the cohort measurements with the reason for exclusion of signals reported below the cartoon. C) Glycosylation profile from three technical replicate measurements of the SILuMAB protein standard in buffer matrix.

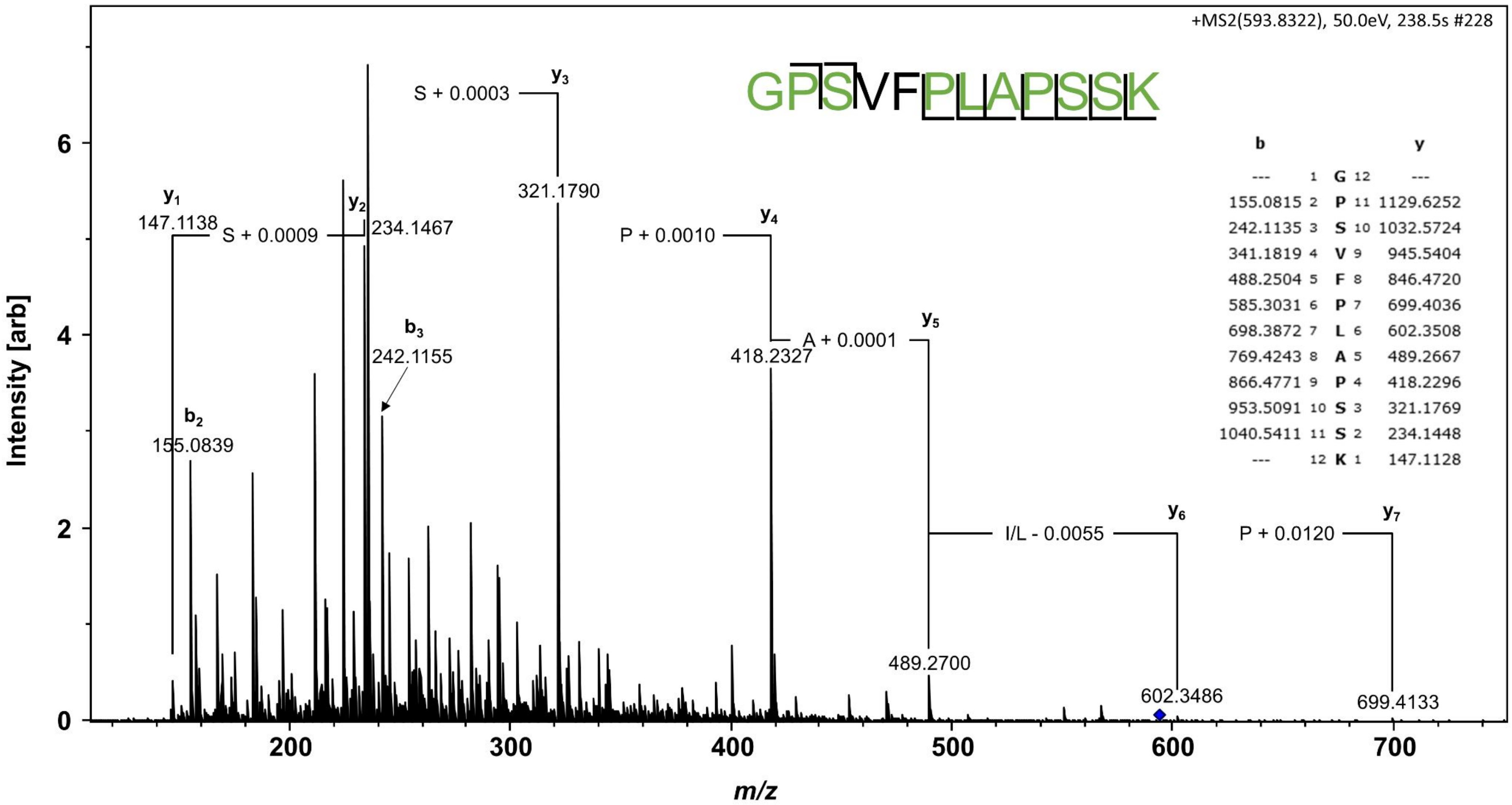

Figure S3: Identification of IgG1 proteotypic peptide. Distances calculated with DataAnalysis (Bruker Daltonics, Bremen, Germany). Theoretical values taken from https://prospector.ucsf.edu/prospector/cgi-bin/msform.cgi?form=msproduct.

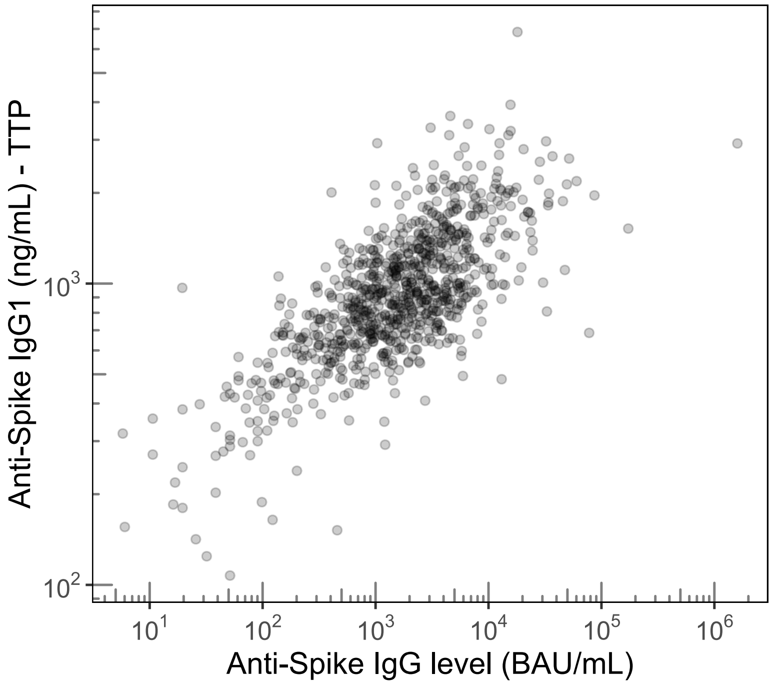

Figure S4: Anti-S IgG1 concentrations based on the TTP peptide plotted against the Luminex® anti-S IgG levels. Only samples yielding a value in both methods were included. $\boldsymbol{r}$ = 0.69 ($\boldsymbol{p}$ < 0.001). $\boldsymbol{n}$ = 844. BAU = Binding Antibody Units.

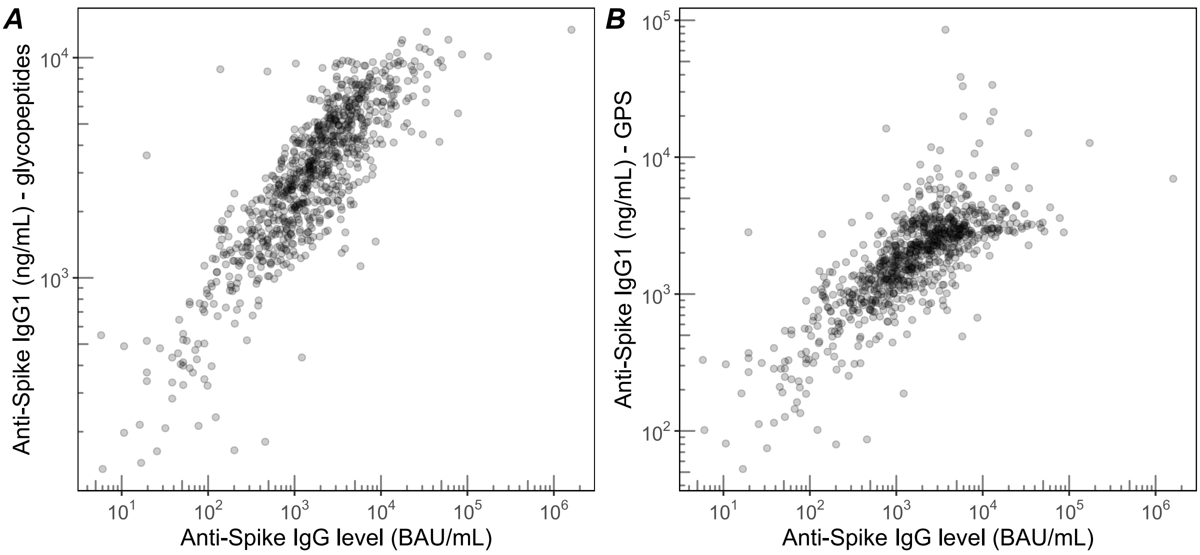

Figure S5: (*A*) Anti-Spike IgG1 concentrations calculated based on the glycopeptides plotted against the Luminex® anti-Spike IgG levels. $\boldsymbol{r}$ = 0.84 ($\boldsymbol{p}$ < 0.001). $\boldsymbol{n}$ = 844. (*B*) Anti-Spike IgG1 concentrations calculated based on the proteotypic peptide GPS. $\boldsymbol{r}$ = 0.76 ($\boldsymbol{p}$ < 0.001). $\boldsymbol{n}$ = 844.

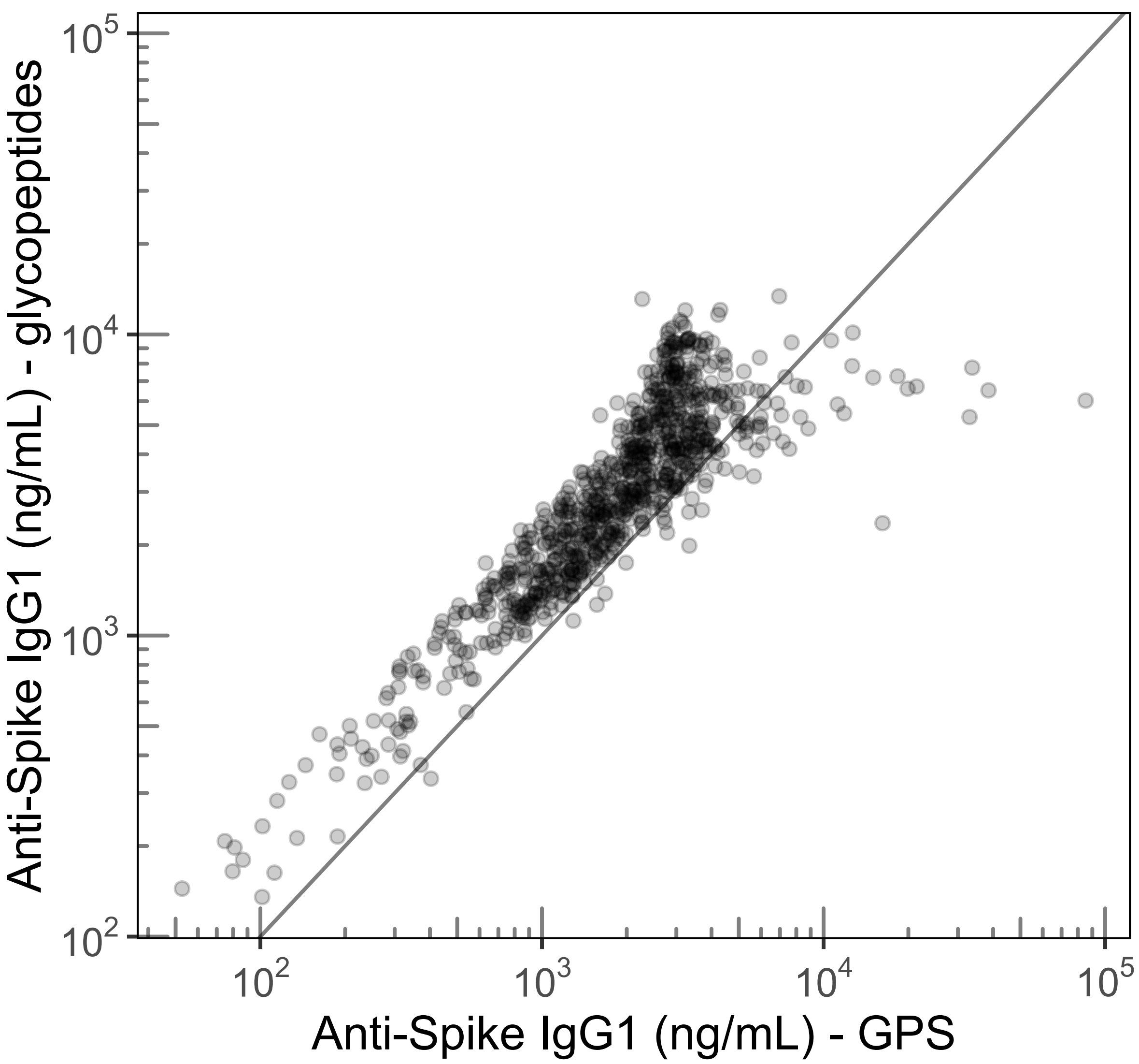

Figure S6: GlYcoLISA anti-S IgG1 concentrations calculated based on glycopeptides correlate strongly with those based on the proteotypic peptide GPS. $\boldsymbol{r}$ = 0.88 (p < 0.001, n = 844). A line of equality $(\boldsymbol{y = x})$ is shown for comparison of the two quantitations.

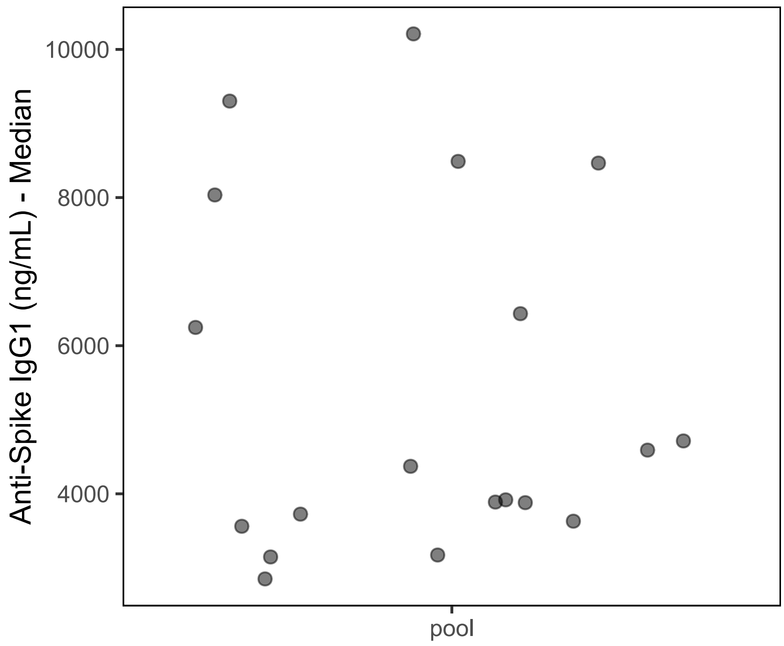

Figure S7: Intermediate precision of IgG1 quantitation and utility of correction. A) Anti-Spike IgG1 concentrations (ng/mL) in the EMC pool samples (N = 19). Mean = 5402 ng/mL and RSD = 44%. One outlier was removed after performing a Grubb's test.

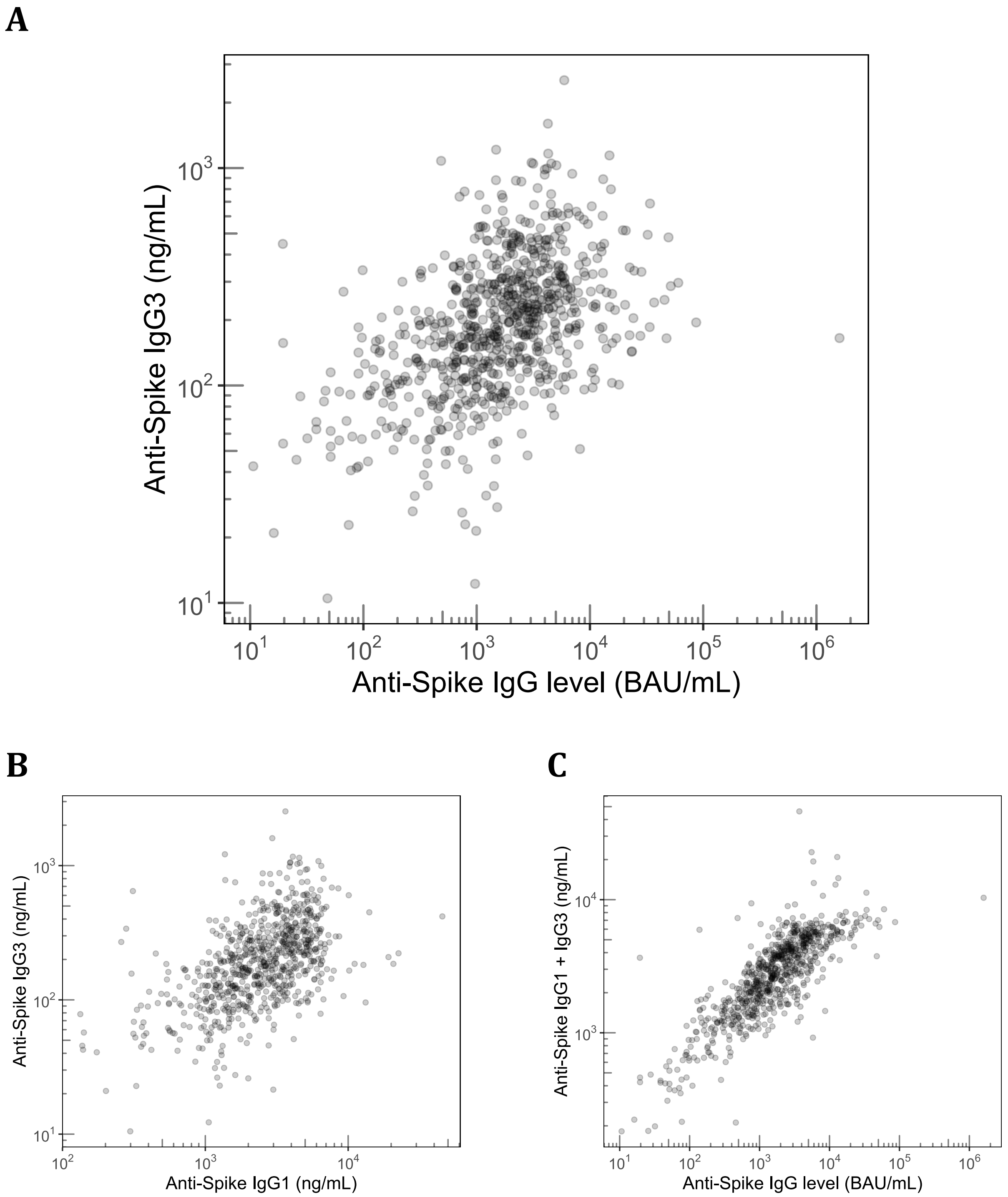

Figure S8: IgG3 quantitation. A) Correlation of IgG3 with Luminex® levels ($\boldsymbol{r}$ = 0.49, $\boldsymbol{p}$ < 0.001). B) Correlation between IgG1 and IgG3 concentrations as assessed by our method ($\boldsymbol{r}$ = 0.54, $\boldsymbol{p}$ < 0.001). C) Correlation between the sum of IgG1 and IgG3 concentrations with Luminex® levels ($\boldsymbol{r}$ = 0.81, $\boldsymbol{p}$ < 0.001).
IgG3 analytes: H3N4F1, H4N4F1, H4N4F1S1, H4N5F1, H5N4F1, H5N4F1S1
